# The geo domain: a review on the conceptualization of geographical and geopolitical entities

**DOI:** 10.3389/fpsyg.2024.1389581

**Published:** 2024-07-10

**Authors:** Ilenia Falcinelli, Chiara Fini, Claudia Mazzuca, Anna M. Borghi

**Affiliations:** ^1^Department of Psychology, Sapienza University of Rome, Rome, Italy; ^2^Department of Dynamic and Clinical Psychology, and Health Studies, Sapienza University of Rome, Rome, Italy; ^3^Institute of Cognitive Sciences and Technologies, Italian National Research Council, Rome, Italy

**Keywords:** geographical concepts, geopolitical concepts, conceptual variability, linguistic relativity, free-listing tasks, ethnophysiographic methods

## Abstract

Investigating how people represent the natural environment and abstract it into geographical (e.g., *mountain*) and geopolitical (e.g., *city*) categories is pivotal to comprehending how they move and interact with the places they inhabit. Yet, the conceptualization of geographical and geopolitical domains has received scant attention so far. To deal with that, we reviewed 50 articles tackling this topic. Most studies have focused on assessing the universality of these concepts—especially geographical ones—mainly using free-listing and ethnophysiographic methods. Current perspectives tend to favor a non-universalistic characterization of these kinds of concepts, emphasizing their high cross-linguistic and cross-cultural variability, especially when compared to other semantic domains. Since geographical and geopolitical features are not pre-segmented by nature, the role of categories imposed by humans is crucial for these concepts. Significantly, their variability does not only depend on “cross” differences: evidence suggests that the cognitive demand requested by the task, idiosyncratic characteristics of individuals such as expertise level, and the typology of inhabited environments are further factors impacting the conceptual flexibility of these domains. Exploring the factors influencing our understanding of geographical and geopolitical categories can provide valuable insights for instructing effective communication policies to enhance sustainable development and address ecological emergencies, taking into consideration diverse cultural backgrounds within different populations.

## 1 Introduction

Conceptualization can be defined as “the process of abstracting the real world into the concepts we use to refer to what is there” (Williams et al., 2012, p. 51). Concepts are the “glue” that connects our past, present, and future (Murphy, 2002). They can be considered cognitive and mental representations of categories we form to comprehend, navigate, and interact with the physical and social environment.

Understanding how people conceptualize the natural world and use geographical and geopolitical categories is becoming increasingly urgent considering the impact on our daily lives of environmental transformations stemming from the climate change emergency and international political contrasts. Unfortunately, although several studies have explored the impact of the ecological crisis on human wellness, focusing, for instance, on emotional (eco-anxiety - Clayton, 2020; Pihkala, 2020) or physical eco-consequences (e.g., Abraham et al., 2023) and identified factors leading pro-environmental behaviors (Li et al., 2019) and strategies to incentivize them (Schultz, 2014; Tam et al., 2021), only a few studies focus on how people represent ecology-related phenomena (e.g., Baquiano and Mendez, 2015, 2016; Falcinelli et al., 2023; Mbaye et al., 2023; Dézma et al., 2024). In addition, scarce attention is dedicated to investigating concepts referring to places in which climate change events typically occur—i.e., geographical kinds such as *mountains, oceans, forests*, and geopolitical entities such as *state, city, metropolis*. In our opinion, this is a critical issue to address, considering that human choices and behaviors are influenced not only by emotional, personological, and contextual aspects, but also by people’s perception and representation of reality (Myers, 2007).

To that end, our review focuses on geographical and geopolitical concepts—from now on, “geo” concepts—with the aim of discussing and summarizing the most influential studies carried out on the topic. We start by defining the domain of inquiry and clarifying the method we used to select the papers. We then detail the rationale behind the choice of focusing on geo concepts, and we outline possible shortcomings that might have motivated the scarcity of studies in this area. We then touch upon cross-cultural, cross-linguistic, and interindividual variability from a theoretical and methodological perspective, looking at how this might shape geo concepts. Most studies, indeed, have emphasized the variability of these concepts when compared to other conceptual domains (e.g., Battig and Montague, 1969; van Overschelde et al., 2004; van Putten et al., 2020) and across different languages and cultures—both industrialized and rural—and current positions tend to favor a non-universalistic characterization of them (e.g., Burenhult and Levinson, 2008; Mark et al., 2011; Turk and Stea, 2014). In addition, the cognitive demand requested by specific tasks (e.g., Mark et al., 1999, 2001; Smith and Mark, 2001a,b; Pires, 2005), idiosyncratic characteristics of individuals such as their level of expertise (e.g., Giannakopoulou et al., 2013; Wartmann et al., 2014; Purves et al., 2023), as well as the typology of the inhabited environment (e.g., Williams et al., 2012; Wartmann et al., 2015) further impact geo conceptualization. Thus, geo concepts well-exemplify the flexibility of the human conceptual system (e.g., Barsalou, 1993; Connell and Lynott, 2014; Mazzuca et al., 2021, 2022). The last section of the review discusses how information about the places we live in is stored and organized in our semantic memory (e.g., Tversky and Hemenway, 1983, 1984; Patton, 1995; Lloyd et al., 1996), leveraging studies inspired by the classical theory of hierarchical levels (Rosch and Mervis, 1975; Rosch et al., 1976, 1978).

Importantly, all the reviewed studies have accessed geo-concepts through the words that express them. Results from the reviewed studies are summarized in Table 1.

**TABLE 1:** Synthesis of the studies reviewed in the paper, along with their aims, tasks, procedures, and results.

Study	Aim	Task	Procedure	Results
**Variability of geo concepts (higher than other semantic domains) between individuals sharing the same linguistic and socio-cultural background**				
Battig and Montague, 1969; van Overschelde et al., 2004.	Extending and updating existing category norms for the US population.	Free-listing task.	Students from different US universities were asked to write down as many examples as possible for each presented category. Among target categories, also geographical (e.g., “A Tree”, “A fish”) and geopolitical (e.g., “A City”, “A State”) ones were included.	The authors indirectly provided the first evidence on the variability of geo concepts, finding that geo categories were the only ones whose members were more differently distributed across groups, despite individuals sharing the same linguistic and socio-cultural context. They also found slight differences in results between their studies, which indicated a high “generational” stability of findings.
**Cross-cultural and cross-linguistic variability of geo concepts, higher than other semantic domains**				
Mark and Turk, 2003; Tsosie, 2006; Mark et al., 2007, 2010, 2011; Turk et al., 2011, 2012; Turk and Stea, 2014.	Examining the linguistic encoding of landscape terms in two aboriginal languages—i.e., Yindjibarndi and Navajo—when compared to English.	Ethnophysiographic methods.	The authors extracted Yindjibarndi and Navajo landscape terms from dictionaries and then refined them through discussions with local language experts; they also asked natives to describe pictures illustrating their motherland. Then, they compared the meaning of the extracted words with their English equivalents.	None of the Yindjibarndi and Navajo words aligned in meaning with each other and English, thus supporting the hypothesis that linguistic encoding of landscape features varies across different languages and cultures (ethnophysiographic hypothesis).
Brown, 2008; Burenhult, 2008; Cablitz, 2008; Enfield, 2008; Levinson, 2008; O’Connor and Kroefges, 2008; O’Meara, 2010; O’Meara and Bohnemeyer, 2008; Senft, 2008; Widlok, 2008; Huber, 2014; Mazzitelli, 2018.	Supporting the ethnophysiographic hypothesis, examining the linguistic encoding of landscape terms in different aboriginal languages (e.g., Mayan, Austronesian).	Ethnophysiographic methods.	The authors extracted native landscape terms from dictionaries and compared their meanings with English equivalents.	Results from all studies supported the ethnophysiographic hypothesis, showing a different linguistic encoding of geo features depending on the language and culture.
Burenhult and Levinson, 2008.	Performing a large-scale comparison across all Aboriginal languages of the previous section, assessing their alignment in landscape encoding with each other and English. All speech communities were genetically, typologically, and geographically unrelated.	Ethnophysiographic methods.	Analysis of the linguistic encoding of landscape features in words extracted from local dictionaries and comparison of their meaning with English equivalents.	None of the languages aligned in meaning with its English equivalent, thus confirming the ethnophysiographic hypothesis.
van Putten et al., 2020.	Analyzing the cross-linguistic conceptualization of the term *landscape*, when compared to other two concrete concepts, i.e., *bodily parts and animals*, across seven European languages (Dutch, English, French, German, Italian, Spanish, and Swedish).	Free-listing task.	Participants were asked to list as many examples as possible within 3 min for *landscape, animals*, and *bodily parts* categories.	The *landscape* concept more consistently varied across languages than the other kinds of concepts, thus showing a weaker structural core than the latter.
Striedl et al., 2024.	Performing a follow-up of van Putten et al. (2020) study to investigate cross-cultural differences in *landscape* conceptualization by relying on a different methodology, i.e., a rating task.	Rating task.	Native speakers of three related European languages—English, French, and German—were asked to provide evaluations on the extent to which several exemplars of the *landscape* category (extracted from van Putten et al., 2020) activated sensory, motor, and emotional components.	Results revealed an overall robust alignment in ratings within languages and across speech communities, suggesting a similar conceptualization of *landscape* across linguistic groups. However, cultural experiences also modulated evaluations, particularly in relation to specific terms.
Burenhult et al., 2017.	Analyzing linguistic expressions for the concept of *forest* in six indigenous languages (i.e., Avatime, Duna, Jahai, Lokono, Makalero, and Umpila/KuukuYa’u), compared to English.	Ethnophysiographic methods.	Data was first-hand collected through stimulus-based and elicitation tasks, interviews, and counts of the natural occurrences of salient terms in recordings from language experts.	None of the languages possessed terms comparable in meaning to the English *forest*. Moreover, *forest* terms were distributed along an across-languages continuum of abstractness, spanning from highly specific and concrete “tree-encoding” meanings (e.g., in Umpila/KuukuYa’u, and Lokono languages) to more general, abstract “space” meanings (e.g., in Duna and Jahai languages).
**The main driver leading to the formation of geo categories**				
Brown, 2008; Burenhult, 2008; Burenhult and Levinson, 2008; Mark et al., 2010; Turk et al., 2012; Huber, 2014; Mazzitelli, 2018.	Investigating the drivers leading to the formation of geo categories.	Ethnophysiographic methods.	The authors extracted native landscape terms from dictionaries and compared their meanings with English equivalents.	Results revealed the presence of three main drivers: (I) the salience of geographical features, from both a perceptual and cognitive perspective; (II) utilitaristic affordances, i.e., the benefits that a particular place brings to the life of a community;(III) cultural and linguistic models of a group.
Regier et al., 2016.	Investigating the drivers leading to the formation of geo categories. They hypothesized that different ways to categorize the landscape could be the effect of adapting languages to the physical environments where they are spoken. They tested this hypothesis by analyzing the encoding of *snow/ice* terms in different speech communities from cold and warm countries.	Ethnophysiographic methods.	Analysis of multiple sources of data, such as reference works, Twitter, and large digital collections of linguistic and meteorological archives.	The need for efficient communication was shown to shape language. Indeed, the authors found that languages with separate terms for *ice* and *snow* were spoken in both cold and warm regions. By contrast, languages that collapse this distinction were spoken exclusively in warm regions, probably because these could have less pressure—dictated by less practical needs—to preserve the distinction than the former.
O’Meara and Bohnemeyer, 2008.	Investigating the drivers leading to the formation of geo categories. They hypothesized that how the landscape is linguistically encoded might also be affected by the structural properties of a language.	Ethnophysiographic methods.	Analysis of the linguistic encoding of landscape features extracted from Seri aboriginal language dictionaries and comparisons with their English equivalents.	The authors found that Seri’s lexicon was more complex than English and that Seri people linguistically defined landscape features primarily according to their material composition plus other features (e.g., shape, orientation). The complex linguistic system of Seri might lead native speakers to pay more attention to the material and spatial properties of landscape entities compared to English speakers, and this might be due to the structural properties of the language rather than to a specific way of perceiving landscape.
**The influence of task manipulation and expertise on geo conceptualization**				
Mark et al., 1999, 2001; Smith and Mark, 2001a,b.	Exploring how people conceptualize geo concepts and whether experimental manipulations might affect them.	Free-listing task.	US University students were asked to list examples for several geographical categories slightly variable in their formulation, i.e., “a kind of geographic feature/object/concept”, “something geographic”, and “something that could be portrayed on a map”.	Responses to the different phrasings significantly diverged, suggesting that base nouns (e.g., “feature”, “object”, “concept”, “something”) combined with the term “geographic” activated different superordinate categories.
Pires, 2005.	Replicating studies from Mark’s lab (previous section) with a different sample of Portuguese participants, introducing just slight methodological variations.	Free-listing task.	Portuguese University students were asked to list examples for the geographical categories in Smith and Mark, 2001a,b—i.e., “a kind of geographic feature/object/concept”, “something geographic”, and “something that could be portrayed on a map”—plus for a further category taken from Battig and Montague (1969)—i.e., “a natural earth formation”. Example productions were then compared between Portuguese and US groups.	Portuguese and US participants mostly produced common elements for all six categories, showing low variability of geo concepts across countries. They also replicated previous findings from Mark’s lab, confirming a strong effect of experimental manipulation on the kind of word production.
Giannakopoulou et al., 2013.	Exploring the effect of individuals’ expertise on geo knowledge on its conceptualization.	Free-listing task.	Greek geography experts and non-experts were asked to list examples for the geographical categories in Smith and Mark, 2001a,b—i.e., “a kind of geographic feature/object/concept”, “something geographic”, and “something that could be portrayed on a map”. Example productions were then compared between expertise groups.	As in previous studies, they found significant differences in example productions between the experimental manipulations but no substantial effect of expertise.
Purves et al., 2023.	Exploring the effect of individuals’ expertise on geo knowledge on its conceptualization.	Rating task.	German and English geography experts and non-experts were compared based on their knowledge of 25 concepts related to water bodies by collecting sensory, motor, and affective ratings.	Experts’ and lay people’s conceptualizations of water bodies broadly align, while expertise was less relevant for explaining the few differences across language samples.
Wartmann et al., 2014.	Exploring the effect of individuals’ expertise on geo knowledge on its conceptualization.	Ethnophysiographic methods.	The authors investigated the linguistic encoding of plant features in the Madidi aboriginal language, comparing native speakers with the current standard scientific classification of plant terms (e.g., in dictionaries).	Their findings evidenced a gap between the Madidi native conceptualization and scientific taxonomies. Specifically, how the Madidi population classifies vegetation encompassed more numerous and complex terms and reflected more fine-grained differences than those included in the encyclopedia. Results thus showed a divergence between commonsense and scientific geo conceptualizations.
**The influence of places and their familiarity on geo conceptualization**				
Wartmann et al., 2015.	Investigating whether words listed for geographic categories vary depending on the setting where the task is performed.	Free-listing task.	Participants were tested in three different settings, two similar (mountain sites) and one different (city park site) for landscape conformation, and asked to produce all the words that came to their minds starting from the question: “What is there for you in a landscape?”.	The place where the task was performed influenced listed words and their order: participants produced similar terms when they were in similar landscapes and different terms when they were in different places. The task location also affected the memory search strategies of participants: individuals first produced visible elements of the surrounding landscape and then used the memory of a familiar place to name terms for that landscape.
Williams et al., 2012.	Demonstrating that places’ familiarity also plays a role in their conceptualization.	Ethnophysiographic methods.	Participants inhabiting two different areas of Portugal, characterized by a different landscape conformation, were recruited. Participants were asked to watch videos displaying the two regions, name all the landforms they could identify, and provide place names for any locations they recognized.	Differences in the landform vocabulary size and content between the two groups were found, with familiarity mostly accounting for these differences. Participants used more landform terms to describe the most familiar landscapes. In addition, the landform vocabulary content was more detailed when it concerned prominent landscape features in which participants lived. Finally, the number of scenes people recognized positively correlated with the number of landform terms they used to describe the videos in both groups.
**Hierarchical levels of categorization of the geo domain**				
Patton, 1995.	Investigating the hierarchical levels of organization of the geo-information.	///	///	He proposed a three-level hierarchical organization inspired by Rosh’s theory (Rosch and Mervis, 1975; Rosch et al., 1976, 1978), in which geo-information is stored at a subordinate, basic, or superordinate level. The nodes in the subordinate level correspond to actual places a person has experienced (e.g., *Rome*), while the basic-level nodes represent more abstract categories (e.g., *city*). In contrast, at the highest hierarchical level (i.e., superordinal), there is the most abstract spatial concept, i.e., “*place*”.
Lloyd et al., 1996.	Extending Rosh’s theory on hierarchical levels of categorization (Rosch and Mervis, 1975; Rosch et al., 1976, 1978) to geo concepts and exploring the *amount* of information deposited at the superordinate, basic, and subordinate levels.	Free-listing task.	US student participants were asked to list as many characteristics, activities, or parts they could associate with a particular geo term. Categories were superordinate (e.g., “place”), basic (e.g., “city”), and subordinate (e.g., “home neighborhood”).	In line with Rosch’s view (Rosch and Mervis, 1975; Rosch et al., 1976, 1978), a substantial increase of information between the superordinate and basic levels and relatively little differences between the basic and subordinate levels for all three types of information were found.
Tversky and Hemenway, 1983.	Extending Rosh’s theory on hierarchical levels of categorization (Rosch and Mervis, 1975; Rosch et al., 1976, 1978) to geo concepts and exploring the *amount* of information deposited at the superordinate, basic, and subordinate levels.	Mixed methods.	US student participants were provided with both photographs and verbal descriptions of environmental scenes at three levels of abstraction, and they were required to list attributes, activities, and parts they considered appropriate for each scene.	Scenes representing superordinate categories (“indoor” and “outdoor”) shared very few attributes, activities, and parts, suggesting that the scenes were very distinctive. Basic-level scenes (e.g., “school” and “beach”) were less distinctive than superordinate categories, i.e., they shared many attributes, activities, and parts, but also more informative, as participants could list significantly more features. Scenes for more specific categories at the subordinate level (“elementary school” and “lake beach”) did not share significantly more properties than scenes representing basic-level categories—suggesting they were only slightly less distinctive than basic-level scenes.
Tversky and Hemenway, 1984.	Extending Rosh’s theory on hierarchical levels of categorization (Rosch and Mervis, 1975; Rosch et al., 1976, 1978) to geo concepts and exploring the *amount* of information deposited at the superordinate, basic, and subordinate levels.	Mixed methods.	US student participants were asked to provide labels for photographs of scenes and to complete sentences such as “The Kingstons furnished their ______ with furniture they built themselves”.	Participants preferred basic-level terms to answer to both tasks. Indeed, they used more frequently basic level terms when labeling photographs of scenes and when completing sentences describing activities performed in scenes, even though more specific or more general terms would have been appropriate too.

## 2 What do geographical and geopolitical concepts refer to?

This review concerns concepts related to the natural environment. While this is a broad conceptual domain, we narrowed our analysis to two classes of concepts following specific selection criteria. We excluded studies focused on the conceptualization of individual natural kinds like animals (e.g., *tiger, bee, cat*) and plants (e.g., *olive tree, flower, oak*) since these domains have already been extensively investigated (e.g., ethnobiology and folk biology studies - see Medin and Atran, 2004; Atran and Medin, 2008). Literature on ecological concepts—i.e., concepts referring to the ecological emergency (e.g., *climate change, ozone hole, deforestation*)—is still limited (for a few exceptions, see Baquiano and Mendez, 2015, 2016; Falcinelli et al., 2023; Mbaye et al., 2023; Dézma et al., 2024), preventing us from including this class of concepts in the review.

Our review focused instead on geographical and geopolitical concepts, i.e., concepts indicating locations where people usually live and maintain social relations. Specifically, geographical concepts (e.g., *mountains, forests, oceans*) refer to large-scale physical entities and places in which human intervention has been minimal over time, thus remaining well-preserved in their original forms (i.e., the “totally natural” categories from Mausner, 1996; see also Pasca et al., 2018). Conversely, geopolitical concepts (e.g., *cities, states, metropolis*) include geographical elements highly affected by human intervention, such as urbanization, and politically relevant entities (i.e., the “non-natural” categories from Mausner, 1996; see also Pasca et al., 2018).

In our review, we will use the term “geo” to refer jointly to both classes, as in most of the studies we targeted, these were treated together. Instead, we will use specific labels (i.e., geographical and geopolitical) when illustrating studies explicitly focusing on one of the two domains. Notably, the definition we just provided for environmental places—based on the criteria of the degree of human intervention in transforming the natural environment—and their distinction into geographical and geopolitical concepts pertains to a specific discipline and research field, i.e., psychology and psycholinguistics (Mausner, 1996; Pasca et al., 2018). In other fields, such as geography, but also political science, and sociology, the investigation of geographical and geopolitical concepts happens jointly, and their distinction does not exist or is not so marked. The divergence in definitions across disciplines is, *per se*, informative since it further testifies to the variable character of the geo domains, which does not only concern the linguistic and cultural differences, as we extensively shown in the course of the review, but also extends to scientific disciplines.

## 3 Method for the selection of papers

To retrieve relevant articles and materials on the conceptualization of geo entities, we conducted an exhaustive literature search between December 2021 and April 2022, with a subsequent bibliographic search integration in July-August 2023 and April 2024 to include more recently published materials.

A first bibliographic search was performed on Google Scholar and PubMed, with a second double-check made on Scopus. In our searches, we used the conjunction of words that refer to geographical and geopolitical entities (e.g., “nature”, “geographical”, “geopolitical”), with labels denoting their specific status (e.g., “concepts”, “categories”, “features”), and/or with terms indicating tasks typically used in categorization research (e.g., “free-listing”, “ethnophysiographic methods”), or research field typically dealing with categorization (“ethnophysiography”, “ontology”, “hierarchical organization”), and/or with words referred to conceptual variability (e.g., “variation”, “flexibility”, “malleability”) and/or with words related to individual characteristics, such as expertise levels (e.g., “folk”, “commonsense”, “expertise”). Specifically, we used the combination of the following terms: (“green” OR “geographical/geography” OR “geopolitical/geopolitics” OR “natural/nature” OR “natural environmental/environment” OR “ecological/ecology”) AND (“categories” OR “concepts” OR “terms” OR “features” OR “categorization” OR “conceptualization” OR “linguistic encoding” OR “features encoding”, “entities”) AND/OR (“linguistic task” OR “semantic fluency (task)” OR “feature listing (task)” OR “free-listing (task)” OR “property generation (task)” OR “feature generation (task)” OR “category norms” OR “rating (task)”, OR “natural language analysis” OR “ethnophysiographic method(s)”) AND/OR (“ethnophysiography” OR “ontology” OR “ontological investigation” OR “Geographical Information System/GIS”, “hierarchical organization” OR “hierarchical levels”) AND/OR (“variation” OR “variability” OR “flexibility” OR “conceptual flexibility” OR “linguistic relativity” OR “malleability”) AND/OR (“native theory” OR “folk” OR “commonsense” OR “expertise” OR “proficiency” OR “non-expert” OR “laypeople”).

All terms were searched as keywords within the text and as words belonging to the title and/or abstract. We did not apply any restriction on the publication date range—due to the scarcity of contributions on the topic—and we only considered publications in English.

To increase the likelihood of including as many relevant studies as possible, we conducted a further manual search. We inspected the list of references of the publications we retrieved and searched for relevant materials on the most influential authors’ Google Scholar or Scopus Profile.

The literature search yielded a total of 116 papers. Materials consisted of papers in peer-reviewed journals, chapters of books, and unpublished Ph.D dissertations.

**FIGURE 1:**
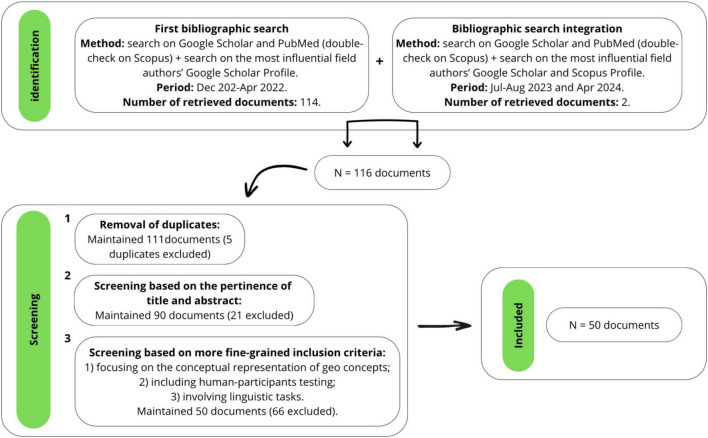
The flowchart shows the steps implemented to select papers presented in this review.

To evaluate the relevance and eligibility of articles, we used a hierarchical approach. The total list of papers was first assessed for duplicates. Then, we screened documents based on their title and abstract and excluded those that did not fit our research topic. The remaining articles were examined more in-depth by reading the entire manuscript, and those that met the inclusion criteria were included in the review. We selected articles that: 1) focused on the conceptual representation of geo concepts—i.e., geographical and geopolitical concepts—rather than on that of other natural entities, like plants and animals, or ecological issues; 2) included studies with designs and results based on human-participants testing rather than computational models; 3) involved linguistic tasks (e.g., ratings, free-listings, ethnophysiographic methods), to avoid potential confounding factors derived by heterogeneous tasks’ requests. Based on such criteria, we excluded 66 documents, thus resulting in a total of 50 articles eligible for the present review. Figure 1 shows a flowchart of the steps we implemented to select the final set of papers we will present in the review.

## 4 Reasons why it is important to study geo concepts

Understanding how people conceptualize geographical entities, such as *mountains, seas, forests*, and geopolitical ones, such as *states, regions, and boundaries*, is crucial for several reasons.

Before illustrating them, it is important to mention some research lines that characterize literature on concepts and categorization and that are relevant to the present review. Among these, some studies have focused on general dimensions that might differentiate concepts, like their degree of concreteness∼abstractness (e.g., Villani et al., 2019), i.e., to what extent concepts evoke the five senses and refer to specific, spatially bounded objects (Borghi and Binkofski, 2014). On top of that, some researchers started to study the domains for which this distinction might be problematic—because it might shift depending on the context (e.g., gender: Mazzuca et al., 2020, 2023; olfaction: Majid et al., 2018). A further important line of research is represented by studies assessing conceptual content and focusing on properties of specific conceptual domains. For example, much behavioral, neuropsychological, and neuroscientific research has been dedicated to investigating differences in the processing and brain representation between living and non-living entities (for reviews: Warrington and Shallice, 1984; Humphreys and Forde, 2001; Forde and Humphreys, 2005), or artifacts and natural kinds (e.g., Keil, 1992), and more recently on concepts that are a sort of hybrid between artifacts and natural objects, i.e., food concepts (Rumiati and Foroni, 2016; Papies et al., 2020; Mazzuca and Majid, 2023). These research strands are worth mentioning as they are the ground for substantiating the claim that geo concepts might be considered hybrid concepts, too (see section “6.3 Two paradigmatic cases of geographical variability: the concepts of *Landscape* and *Forest*”).

What are the reasons that make studying geo concepts particularly important?

First, it might contribute to understanding how people act on and interact with the natural and social environments. Specifically, it could illuminate how people live inside environmental transformations caused by climate changes and political contrasts.

Second, investigating geo concepts is paramount because of their specific cognitive profile. As we will show during the review, these concepts refer to large-scale physical elements. Still, because geographical and geopolitical features are not pre-segmented by nature, the role of categories imposed by humans is pivotal to defining them. Consequently, the geo conceptualization might be more variable than that of other semantic domains (Mark et al., 2010; van Putten et al., 2020), thus representing an excellent example of conceptual flexibility (e.g., Barsalou, 1993; Connell and Lynott, 2014; Mazzuca et al., 2021, 2022).

Two further reasons make the study of geo concepts particularly important. From a scientific standpoint, examining concepts either grounded in physical reality or determined by cultural and linguistic differences could facilitate the construction of an ontology of the geographic and geopolitical domains. In addition, research in this area also has a practical slant. Defining an ontology of the geo domains is fundamental for supporting non-specialist use of Geographic Information Systems (GIS), like *Google Earth*, that assist human activities such as navigation, resource management, and emergency services (Kuhn, 2001). GIS are usually accessed by heterogeneous groups of people with different sociocultural and linguistic backgrounds, interests, and age-related specificities. Hence, addressing the question of the universality of geographic and geopolitical concepts is crucial when dealing with GIScience (Mark and Turk, 2003; Burenhult and Levinson, 2008; Turk and Stea, 2014). To maximize their performance, GIS should account for the substantial variability in encoding geographical and geopolitical features in different languages and cultures. For instance, *Google Earth* maps names of places worldwide, risking forcing indigenous names to a universal ontology derived from an English conceptualization of landscapes (see also Blasi et al., 2022). Locals may carve up reality in quite different ways, evidencing the limits to the efficient use of these tools (Mark and Turk, 2003; Burenhult and Levinson, 2008; Turk and Stea, 2014). In the framework of contrasting the climate change crisis, this understanding can provide a conceptual basis on which to identify and implement more idiosyncratic policies aimed to take actions to contrast climate change consequences and enforce policies for sustainable development, leveraging on how people of different cultures conceive the environment surrounding them. In addition, it can contribute to facilitating the revitalization of global partnerships for sustainable development, considering cultural and linguistic differences (e.g., see Goals 13, 14, 15, and 17 of the United Nations 2030 Agenda for Sustainable Development - United Nations, 2015).

## 5 Reasons why it is difficult to collect data on geo concepts

Despite their importance, gaining knowledge about geographical and geopolitical concepts is quite demanding. In particular, the intrinsic characteristics of these domains make their study particularly challenging.

First, people conceptualize geographical and geopolitical entities ambiguously, both in terms of objects (“what” concepts) and places (“where” concepts - Burenhult and Levinson, 2008). This aspect has been thoroughly examined by scholars who analyzed the encoding of “what” and “where” concepts in different aboriginal languages, such as Makalero (a Papuan language of East Timor - Huber, 2014, 2018), Marquesan (an Austronesian language spoken on the Marquesas Islands - Cablitz, 2008), and Lokono (an Arawakan language of the Guianas - Rybka, 2015, 2016). These studies revealed that “what” nouns typically denote individuated, discrete (Lyons, 1977, p. 693), relatively small and potentially moveable entities (Talmy, 1972, 2000, p. 315), such as *Persons, Animals* and *Physical Objects* (Lyons, 1977) that are typical exemplars of the superordinate “Thing” category (e.g., *chicken, blanket, stone, or John* - Huber, 2018, p. 478; Mackenzie, 1992, p. 256). Conversely, “where” concepts commonly denote entities that can be defined as large and stationary, such as *Artificial* and *Natural Locations* that are common exemplars of the superordinate “Place” category (e.g., *office, church, mountain, cliffs* - Huber, 2018, p. 478–480; Stolz et al., 2014, p.42). While in most cases, the referents of “what” nouns possess clear physical boundaries (Lyons, 1977; Jackendoff, 1983; Langacker, 2013), the referents of “where” nouns mostly lack clear perceptual boundaries that are readily identifiable (Cablitz, 2008; Rybka, 2015, 2016).

In this framework, an interesting perspective on geo concepts came from Lyons (1977, p. 693), according to whom artificial and natural locations might grant a special status of indeterminacy between the “what” and “where” categories, allowing for the possibility of being treated as one or the other in different languages. Take, for example, the concept of *forest*. In the sentence “The forest is huge”, the *forest* is perceived as an object located on the surface of the Earth. On the contrary, in the sentence “The ruin is in the forest”, the *forest* becomes a place, i.e., a part of the Earth’s surface where we move (Lyons, 1977, pp. 477). Likewise, the concept of the *city* can be conceived both as an object we talk about (e.g., “This city is called Rome”) and as a location (e.g., “About 40 thousand people live in that city”). Therefore, the same geo concept can belong to different ontological categories depending on the meaning arbitrarily assigned to it (Jackendoff, 1983; Landau and Jackendoff, 1993), hence displaying different conceptual properties.

To integrate this evidence, Stolz et al. (2014) showed that “Places” objects (e.g., *office, school, church*, Stolz et al., 2014, p. 42) were the only nouns that did not need an overt locative marker (“zero-marking” - Huber, 2018) to be identified as places—thereby representing intrinsically “where” nouns, i.e., nominal predicates with an intrinsic locative interpretation. Moreover, it was found that zero-marking of natural location nouns was a typical pattern across languages—it was present in several investigated aboriginal languages (Stolz et al., 2014; Huber, 2018). In contrast, “what” concepts (i.e., those referring to “Thing” objects such as *chicken, blanket, stone, or John* - Mackenzie, 1992, p. 256) can be used only with semantically specific locative verbs to denote locations since they do not have an intrinsic “where” interpretation.

To summarize, concepts like *mountains, lakes*, and *cliffs* assume a double ontological status—both of object and places—while this is not the case of other conceptual domains.

The lack of clear perceptual boundaries of natural and artificial locations has a further implication related to the criteria we use to determine entity boundaries, making studying these domains particularly hard. Geographical entities such as *valleys, mountains*, or *lakes* are parts of the Earth’s surface, which is an objectively continuous surface (Smith and Mark, 2001a, p. 93). Differently from plants and animals, landforms more properly belong to continua (Mark et al., 2010). Due to this, landscape features are not, for the most part, pre-segmented by nature but by human categorization processes. In other words, the lack of clear perceptual boundaries for natural entities makes human decisions rather than perception the most salient criterion to determine their status. For example, we can think of a *valley* in different ways, e.g., “as a concave fold between mountain ranges, or as the flat bottom of such a fold, or as the entire drainage area right up the flanks or do so without any such concept” (Burenhult and Levinson, 2008, pp. 137–138). Similarly, boundaries of artificial entities such as *states, cities, and nations—*despite being often physical like walls, water bodies, and fences—do not pre-exist; they are usually defined by political conventions. A direct consequence is that geographical and geopolitical concepts might be more variable across individuals and more affected by contextual factors such as language, culture, environment, and individual perspectives than other domains (Mark et al., 2010). In line with this, for instance, research has shown that different languages segment and outline landforms differently (e.g., Burenhult and Levinson, 2008; see section 6.3 “Two paradigmatic cases of geographical variability: the concepts of *Landscape* and *Forest*”).

Finally, the strong interconnection and mutual influence between the geographical and geopolitical domains makes it hard to disentangle them sharply and thus study them separately. Take, for example, national borders. Political forces usually define them, but natural features can also contribute by providing physical limits. For instance, the water surrounding the land could induce governments to decide that “Italy” ends where the sea starts. Likewise, political interests can transform a geographical element like a *valley* into a geopolitical entity such as a *city* or, similarly, a *coast* into a *seaport*. While the physical referent is the same, the concept endorses different ontological statuses depending on human interventions.

To sum up, the ambiguous nature of geographical and geopolitical concepts, their high variability, and their peculiar relationship with each other and their referents make their study particularly complex. We argue that these aspects should be considered to make cross-cultural communication more efficient and enhance governmental policies aimed at increasing sustainability in different countries.

## 6 Cross-linguistic and cross-cultural variability of geo concepts

Whether speaking different languages differentially shapes human cognition is still debated in cognitive science (Lucy, 1997; Casasanto, 2008; Whorf, 2012; Majid et al., 2018; Blasi et al., 2022). Relativist accounts contrast with nativist ones, according to which cognitive processes remain invariant regardless of specific languages and cultures. While several studies showed that language has a pervasive influence on cognition (Malt and Majid, 2013; Goddard and Wierzbicka, 2014; Kemmerer, 2019, 2022), such an influence might be more marked for some domains. It has been proposed elsewhere (Borghi and Binkofski, 2014; Borghi et al., 2018a,b, 2019a,b; Borghi, 2019, 2023) and presenting some data showing (Borghi and Mazzuca, 2023; Da Rold et al., 2023) that linguistic variability might be more pronounced for abstract than for concrete concepts. In the context of the debate between relativists and universalists, geo concepts might represent an interesting case. Indeed, several studies have demonstrated that their conceptualization—and in particular that of geographical entities such as landscape, water bodies, and other geographical features—varies more across languages and cultures than other conceptual domains (such as animals or body parts - see van Putten et al., 2020). Researchers have addressed this issue using a multitude of approaches. Two methodologies have dominated this literature so far, namely free-listing tasks and methods from ethnophysiography. Free-listing tasks are semantic fluency tasks that collect the frequency and type of properties generated for specific categories of concepts (e.g., Battig and Montague, 1969; van Overschelde et al., 2004; van Putten et al., 2020). Instead, ethnophysiographic methods analyze the encoding of geographical features in natural languages (e.g., Mark et al., 2010; Turk et al., 2012).

In the research field we examined, free-listing was employed to study the categorization of geographical and geopolitical entities, while ethnophysiographic methods primarily focused on geographical ones.

In the following subsections, we will first separately focus on these two methodologies and the geo-flexibility results they yielded (sections “6.1 The variability of geo concepts: evidence from free-listing tasks” and “6.2 The variability of geo concepts: evidence from ethnophysiographic methods”). We will then report evidence on conceptual variability for two geo concepts, i.e., *landscape* and *forest* (section 6.3 “Two paradigmatic cases of geographical variability: the concepts of *Landscape* and *Forest*”). Finally, we will discuss the reasons that underlie the emergence, variable in nature, of geo concepts (section 7).

### 6.1 The variability of geo concepts: evidence from free-listing tasks

Free-listing is a particular kind of semantic fluency task. It consists of an elicitation method requiring individuals to list examples for named categories such as *food items*, *colors*, or *geographic features*. The goal of free-listing is to define salient elements of a specific cognitive domain for a target population (Berlin and Kay, 1991; Smith and Mark, 2001a,b; Bernard, 2006; Hough and Ferraris, 2010), identifying the instances of a given category that a cultural group more commonly produce to exemplify the category or to illustrate its characteristics.

Employing this methodology, Battig and Montague (1969) indirectly shed the first light on the sociocultural variability of the geo domain. Their pioneering work aimed at updating and expanding the Connecticut category norms, originally collected and published by Cohen et al. (1957), and it was based on what they called the “elicitation-of-example procedure”. Specifically, the authors asked 442 students from two US universities (the University of Maryland and the University of Illinois) to produce examples for 56 categories of concepts, writing down as many items as possible in 30 seconds. Among target categories, geopolitical (e.g., “A City”, “A State”) and geographical objects were also included. However, geographical categories included terms referring to single natural elements like plants and animals (e.g., “A Tree”, “A fish”, “A Snake”). Their results revealed that overall correlations between the frequencies of listed exemplars by the two samples for each category were globally high, providing impressive evidence for the generalizability of these norms. Notably, geographical and geopolitical were the only categories constituting an exception. Indeed, their members were the most variable, as revealed by Pearson’s correlations falling below .90—while the average correlation coefficient for all the other categories was .96—, thus showing a highly different distribution across the East and Midwest.

A subsequent work by van Overschelde et al. (2004) further confirmed these findings. Their study aimed to expand Battig and Montague (1969) and collect new normative data that could account for the cultural changes occurring since 1969. Using the same method as the original study, the authors broadened the sample, targeting participants from three US universities (University of Colorado, University of Maryland, and University of North Carolina). Considered categories were mainly identical to those of Battig and Montague (1969), but van Overschelde et al. (2004) also added 14 new categories (e.g., “A type of car”, “A Herb”). Overall, results replicated previous ones, revealing a high “geographical stability” of norms across countries, with high Pearson’s correlations—above .90—for all the categories except, again, for geo concepts. In addition, the new and old sets of norms aligned quite well, with minimal differences in the average correlations between datasets, thus also showing a high “generational” stability.

All this evidence suggests that geo concepts vary across sociocultural contexts more than other conceptual domains—even within the same country and across people speaking the same language. Interestingly, this trend also remains constant over time.

### 6.2 The variability of geo concepts: evidence from ethnophysiographic methods

Whether geo conceptualization—in particular, geographical—can be considered universal or specific to each culture has been more directly investigated within the domain of ethnophysiography. Ethnophysiography is a recent and promising subfield of *ethnosemantics*. Ethnosemantics investigates how semantic domains are encoded across languages and to what extent their linguistic organization reflects culture-specific *vs* universal principles (Berlin et al., 1974). Ethnophysiography follows the same goals but specifically focuses on the geographical realm. In particular, it deals with sociocultural differences in the human conceptualization of landscape as indicated by the way people speaking different languages talk about and name geographical features using generic terms, toponyms (place names), and idiosyncratic nicknames (Mark et al., 2010, 2011; Turk and Stea, 2014). These linguistic expressions are supposed to reflect the underlying cognitive classification of the domain. The employed methodology is based on comparing the geographical features in different native languages of both industrialized and rural environments—extracted through different methodologies (e.g., from local dictionaries; through interviews with aboriginal people or language experts; asking natives to describe pictures of their landscapes (for more details, see e.g., Mark and Turk, 2004; Mark et al., 2007, 2010; for a review, see Turk et al., 2012). In some cases, these methods allowed the creation of illustrated dictionaries for Aboriginal languages (e.g., Turk and Mark, 2008; Mark et al., 2019).

In their seminal works, Mark et al. (2019) addressed for the first time the universality of geographical concepts by examining landscape terms in two aboriginal languages —i.e., Yindjibarndi and Navajo—respectively, spoken in the rural areas of the Pilbara region of North-Western Australia and in New Mexico/Arizona of South-Western United States (Mark and Turk, 2003, 2004; Mark et al., 2007, 2010; Turk et al., 2011(for a discussion, see Tsosie, 2006; Mark et al., 2011; Turk et al., 2012; Turk and Stea, 2014). The geographic conformation of the two targeted areas was quite similar. However, the cultural background and language spoken by the two communities arose from different sources, providing an excellent opportunity for linguistic comparisons (Turk et al., 2012). Methodologically, the authors selected native terms indicating topographic features (such as convex landforms and water bodies) either from dictionaries and then refining them through discussions with local language experts (Mark and Turk, 2004) or asking natives to describe pictures illustrating their motherland (Mark et al., 2007). Once defined, Yindjibarndi and Navajo terms were compared with each other and their English equivalents. Results revealed that Yindjibarndi and Navajo words did not align in their meaning with each other and English, thus providing strong support to the hypothesis that linguistic encoding of landscape concepts varies across different languages and cultures (Mark et al., 2011; Turk and Stea, 2014).

Further studies provided similar results based on a broad range of aboriginal languages, such as Tzeltal (Mayan, Mesoamerica - Brown, 2008), Jahai (Mon-Khmer, Malay Peninsula - Burenhult, 2008), Marquesan (Austronesian, Polynesia - Cablitz, 2008), Lao (Tai, Mainland Southeast Asia - Enfield, 2008), Yélî Dnye (isolate, Island Melanesia - Levinson, 2008), Lowland Chontal (isolate, Mesoamerica - O’Connor and Kroefges, 2008), Seri (isolate, Mesoamerica - O’Meara and Bohnemeyer, 2008; O’Meara, 2010), Kilivila (Austronesian, Island Melanesia - Senft, 2008), Akhoe Hai (Khoisan, southwestern Africa - Widlok, 2008), Nalik (Astronesian, Papua New Guinea - Mazzitelli, 2018), and Makalero (East Timor, Papua New Guinea - Huber, 2014).

Interestingly, Burenhult and Levinson (2008) performed a large-scale comparison across all these languages, assessing their alignment in landscape encoding with each other and English. The authors relied on the aforementioned existing data, and, notably, all speech communities were genetically, typologically, and geographically unrelated. Once again, the results corroborated the relativistic hypothesis. For example, the English category *valley* was not encoded by all languages. While Kilivila, Lowland Chontal, and Tzeltal had terms comparable to the English *valley*, in Marquesan, the meaning of the word for *valley* also extended to *river* and *village*. In contrast, Lao and Yélî Dnye lacked a *valley* term, with the closest equivalent meaning things like *gradient* or *bottom of inclined plane* (Burenhult and Levinson, 2008).

Overall, this evidence broadens the findings illustrated in the previous section, showing through another methodology how the conceptualization of geo—in this case, geographical—features vary across individuals who do not share the same socio-cultural and linguistic backgrounds.

### 6.3 Two paradigmatic cases of geographical variability: the concepts of *Landscape* and *Forest*

We now report recent studies investigating two lexical categories—*landscape* and *forest*—that can be considered paradigmatic examples of the variability of geo concepts.

Specifically, van Putten et al. (2020) analyzed the cross-linguistic conceptualization of the term *landscape* across seven European languages (Dutch, English, French, German, Italian, Spanish, and Swedish). The authors compared the free-listing production of these speech communities for *landscape* with that provided for two other concrete concepts: *animals* and *body parts*. Free-listing data were collected from over 400 native speakers, who were required to list as many examples as possible within 3 min. Results revealed that, across languages, participants found listing landscape exemplars the most arduous task. Indeed, individuals produced fewer exemplars for *landscape* than for *animals* and *body parts*; in addition, landscape had the least number of shared exemplars and shared co-occurrence pairs across languages and the widest variety of terms among the most cognitively salient exemplars. These findings suggest that the geographical concept of *landscape* more consistently varies across languages than other concepts like *body parts* and *animals*. Data also contradicts the widespread assumption that the *landscape*’s conceptualization is universal and homogeneous across speech communities, at least in Europe (Council of Europe, 2000). Contrarily, this concept differs substantially across languages, showing a weaker *structural core* than other conceptual domains (van Putten et al., 2020).

Along these lines, Striedl et al. (2024) performed a follow-up of this study to investigate cross-cultural differences in *landscape* conceptualization through a rating task. Specifically, the authors asked 289 native speakers of three related European languages—English, French, and German—to evaluate the extent to which several exemplars of the *landscape* category (extracted from van Putten et al., 2020) activated sensory, motor, and emotional components. Results revealed an overall robust alignment in ratings within languages and across speech communities, suggesting a similar conceptualization of *landscape* across linguistic groups. However, cultural experiences also modulated evaluations, as highlighted by differences in ratings for some sensorimotor (e.g., smell, hearing, vision) and emotional (e.g., valence, arousal) associations, particularly in relation to specific terms. This evidence highlights that the domain of *landscape*, is conceptualized similarly across similar speech communities, even if some dissimilarities also exist.

Another interesting example of cross-linguistic variability of geo concepts comes from the investigation of the concept of *forest*. International forestry programs often operate under the assumption that individuals similarly perceive, think, and talk about *forests*, conceived as *tree covers*. However, several contributions have demonstrated to which extent this concept varies as a function of language and culture. This is well exemplified by the distinction between the English term *forest* and its closest equivalent in the Lowland Chontal language (Oxaca, Mexico), i.e., the term *muña*. Despite endorsing meanings similar to the English *forest* or *jungle, muña* also covers meanings like *bush, underbrush, overgrown wilderness*, or *any type of weeds or garbage* (O’Connor and Kroefges, 2008, p. 299). Hence, even if *muña* is the Lowland Chontal closest to the English term, the word has a broader meaning aside from *tree cover*, also encompassing the notion of a disorderly environment, which does not necessarily imply vegetation.

Along the same lines, Burenhult et al. (2017) described and analyzed the concept of *forest* in six indigenous languages when compared to English: Avatime (Ghana), Duna (Papua New Guinea), Jahai (Malay Peninsula), Lokono (the Guianas), Makalero (East Timor), and Umpila/KuukuYa’u (Cape York Peninsula). Data were first-hand collected through stimulus-based and elicitation tasks, interviews, and counts of natural occurrences of salient terms in recordings from language experts. A comparative analysis showed that none of such languages possessed terms comparable in meaning to the English *forest*. Interestingly, *forest* terms were distributed along a continuum of abstractness, from highly specific and concrete “tree-encoding” meanings to more general, abstract “space” meanings. For instance, the Umpila/KuukuYa’u terms *maalatha* and *thungkuyu* imply tree cover, but they are more specific than the English equivalent. Umpila/KuukuYa’u language lacks a superordinate *forest* term that does not specify the tree species. This would be equivalent to English, which has two different terms for *coniferous* and *deciduous forest* but no generic term for *forest*. On the other hand, Duna and Jahai have a term carrying a more general spatial meaning of *outdoors, outside*, or *outside realm*, but no reference to vegetation. Interestingly, in languages that conceptualize *forest* more concretely and include vegetation (i.e., Umpila/KuukuYa’u, Lokono, Makalero, and Avatime), referents are perceived as containers with clear boundaries. By contrast, Duna and Jahai conceptualize forests as outside—as opposed to inside—and as infinite rather than bounded.

Overall, the two examples of *landscape* and *forest* show that languages partition geographical concepts differently. This is in line with the hypothesis that, although they typically refer to concrete entities (e.g., *mountain, lake, forest*)—i.e., elements we can experience through actions and senses (e.g., Brysbaert et al., 2014)—geographical concepts also seem to display features that are more typical of abstract concepts—like being consistently variable across languages and cultures (Borghi et al., 2018a,b, 2019a,b; Borghi, 2019, 2022; Borghi and Mazzuca, 2023), thus perhaps showing a *hybrid* character (for similar results for other semantic domains, see Falcinelli et al., 2023; Falcinelli et al., 2024). Furthermore, these examples clearly highlight the importance of considering conceptual and linguistic variability in policies aimed at improving sustainability, such as those of the United Nations 2030 Agenda for Sustainable Development (United Nations, 2015), to make their communication with different populations more effective.

## 7 The main drivers leading to the formation of geo categories

While cross-linguistic evidence suggests geo conceptualizations consistently vary across sociocultural contexts, most languages seem to possess terms that denote geographical features—although partitioning the environment in culture-specific ways. This raises the question about the origins of these mental categories. Burenhult and Levinson (2008) identified three main forces: I) the salience of geographical features, from both a perceptual and cognitive perspective; II) utilitaristic affordances, i.e., the benefits that a particular place brings to the life of a community; and III) cultural and linguistic models of a group (see also studies in ethnobiology for similar proposals: Hunn, 1982; Berlin, 1992; Malt, 1995). The weight of these factors could diverge across domains (Huber, 2014; Mazzitelli, 2018).

Through the analysis of Indigenous languages, several studies in ethnophysiography unveiled the importance of these three aspects as milestones for categorizing (e.g., Brown, 2008; Burenhult, 2008; Mark et al., 2010, Turk et al., 2012; Huber, 2014; Mazzitelli, 2018). For instance, Mazzitelli (2018) compared English with Nalik, an Austronesian language spoken in the New Ireland Province (Papua New Guinea), and found that Nalik’s categorization of landscape features was tightly linked to their affordances (i.e., action opportunities - Gibson, 1977): for instance, *daanim* “freshwater” (and by extension “river”, “creek”) and *raas* “salty water” (and by extension “sea”) were not distinguished by size or shape, but exclusively by their chemical properties, and hence by the use the community can make of them (one can drink *daanim* but not *raas*). Along with affordances, perceptual salience—i.e., the visual prominence of landscape features that makes them easily recognizable and perceived as distinct elements (as an elevation as opposed to flat terrain)—also played an important role. For example, the distinction between *raas* (“shallow sea on the reef”) and *laman* (“open sea”) can be traced back to their visual properties (the open sea is deeper and darker than the shallow sea on the reef), as well as to their affordances (in the open sea different fishes are found than on the reef). Finally, the cultural meaning of places and their importance to the community’s life were a further categorization criterion, driving the attribution of proper names to geographical features. For instance, river sources, canoe passages, caves, and villages all had a proper name because, according to Nalik’s beliefs, they were where spirits lived. Instead, even perceptually salient entities like rivers and smaller hills mostly lacked proper names and were referred to using the name of the land they flowed through or were located in Mazzitelli (2018).

Additionally, the likelihood of a feature being easily communicated can further underlie landscape categorization. Regier et al. (2016) proposed that different ways to categorize the landscape are the effect of adapting languages to the physical environments where they are spoken to facilitate efficient communication among native people. They tested this hypothesis by analyzing the encoding of *snow/ice* terms in different speech communities. It has been suggested that Eskimo/Inuit languages possess more terms to indicate different subtypes of snow (e.g., *aput* “snow on the ground”, *qana* “falling snow”) than other languages such as English (Martin, 1986; Whorf, 2012), hence partitioning more sharply the notion of snow compared to other languages—although this idea has been largely questioned by other evidence (e.g., Pullum, 1989, 1991). Regier et al. (2016) empirically tested this hypothesis by analyzing multiple sources of data, such as reference works, Twitter, and large digital collections of linguistic and meteorological archives. They aimed to understand whether geographic position and temperature could predict the presence of different terms for *snow* and *ice* in a given language. Specifically, they predicted a link between warm temperatures and a unique semantic category encompassing *ice* and *snow*. Results aligned with these predictions: languages with separate terms for *ice* and *snow* were spoken in cold and warm regions. By contrast, languages that collapse this distinction in their lexicons were spoken exclusively in warm regions. This asymmetry is consistent with the view that the need for efficient communication probabilistically shapes language. In line with this, there should be less pressure to preserve the distinction—and thus a stronger tendency to collapse the two concepts—in warm than cold countries. Notably, this phenomenon characterizes not only Eskimo/Inuit languages but also other languages spoken in cold climates, such as Russian (Krupnik and Müller-Wille, 2010).

Finally, the specific structural properties of languages can also influence how people think about landscapes. O’Meara and Bohnemeyer (2008) investigated this aspect by comparing English and Seri (Mexico) terms for geographical features. The authors found that Seri’s lexicon was more complex than English and characterized by a prevalence of descriptive-analytical terms. Seri people linguistically defined land and water forms primarily according to their material composition and other features: shape, orientation, spatial, and physical features. O’Meara and Bohnemeyer (2008) proposed that this complex “model” for forming geographical terms might be due to the peculiarity of the Seri language rather than to specific ways of conceptualizing geographic entities. Indeed, complex expressions similar in structure to those for landscape features were widespread in Seri and extended to natural kinds and artifact terms. This, however, does not rule out the possibility that the linguistic structure of Seri landscape terms might have cognitive consequences. Indeed, the analytic linguistic system of Seri might lead native speakers to pay more attention to the material and spatial properties of landscape entities compared to speakers of languages with monomorphemic geographical terms, such as English.

Overall, all these studies suggest that the conceptualization of the geographic domain is not universal, and differences can emerge because of the interplay of different factors. Some point to the importance of perceptual or cognitive salience of geographical features, their action opportunities (i.e., affordances), and human cultural beliefs and models (Burenhult and Levinson, 2008). Others instead emphasize the centrality of communication efficacy in specific environmental settings (Regier et al., 2016). In addition, specific structural characteristics of a language are an additional criterion to consider when drivers of categorization are investigated (e.g., O’Meara and Bohnemeyer, 2008).

## 8 Further factors affecting the variability of geo concepts

While in previous sections we tackled cross-cultural and cross-linguistic divergences in geo conceptualization, here we focus on other possible sources of variability. Specifically, we show how individual levels of expertise, the task’s cognitive demand, the experimental setting, and its familiarity can differentially affect geo conceptualizations.

### 8.1 The commonsense conceptualization of geo concepts and the influence of the type of task on word generation

In this section, we review studies seeking to address how laypeople represent the geo domain, that is, we focus on *folk* conceptualization. Most of these studies leverage free-listing and—to a minor extent—ethnophisiograpic and ratings methods with the implicit assumption that results from these procedures can reflect the organization of people’s knowledge about geographical and geopolitical entities.

The interest in folk conceptualization of the geo domain became particularly pressing at the end of the 20^th^ century due to the availability of geographic information not only for experts but also for heterogeneous groups of laypeople. Nowadays, it appears similarly crucial since promoting wellness and sustainable development involves reaching many people and communicating effectively with them, thanks to considering their peculiar ways of conceptualizing the environment. At the end of the last century, GIS scientists felt the necessity to explore the commonsense conceptualization of the geo domain to allow non-experts a proper understanding and use of geographic information. In this framework, some scholars aimed to develop a theory of native or *folk* geography, reflecting what had already been done in other fields (e.g., commonsense physics: Hayes, 1985; Smith and Casati, 1994; Egenhofer and Mark, 1995). In a pioneristic experiment, Mark et al. (1999) asked participants to list examples of “a kind of geographic feature” under controlled conditions. Remarkably, based on the assumptions of academic geography, participants almost entirely generated natural geographic features. *Mountain, river, lake, ocean*, and *hill* were the most often listed exemplars, with *town and city*, i.e., geopolitical entities, hardly listed. In subsequent studies, the authors manipulated the combination of target terms, using linguistic stimuli such as “a kind of geographic feature/object/concept”, “something geographic”, and “something that could be portrayed on a map” (Mark et al., 2001; Smith and Mark, 2001a,b). When presented to different groups of US university students, responses to the different phrasings significantly diverged, suggesting that base nouns (e.g., feature, object, concept, something) combined with the term “geographic” activated different superordinate categories. To illustrate, “geographic feature”, “something geographic”, and “geographic concept” elicited almost exclusively natural features such as *mountain, river, lake, ocean*, and *hill*. Artificial geographic features such as *town* and *city* were rarely listed. The label “geographic object”, instead, induced participants to provide mostly artifacts with a geographical meaning, such as *map, atlas, globe, and compass*. Finally, the phrasing “can be portrayed on a map” elicited primarily geopolitical subdivisions (e.g., *countries, states*) and geographical-scale artifacts (e.g., *roads, cities*).

Pires (2005) replicated these results with Portuguese participants, introducing three minor methodological variations. First, participants were asked to list items for all categories instead of listing exemplars only for one type of label combination. Second, the production was restricted to six examples for each category instead of being illimited. Finally, an additional category was introduced, i.e., “a natural earth formation”, taken from the original work of Battig and Montague (1969), with which items production were compared. The comparison between Pires (2005) and previous results (Battig and Montague, 1969; Smith and Mark, 2001a) revealed remarkable similarities in laypeople’s conceptualization of the geographical domain. Indeed, Portuguese and US participants produced many common elements for all six categories. To illustrate, they all tended to list geographical features such as *rivers* and *mountains* for all the categories, except for the label “something that could be portrayed in a map”, which encompassed more artifactual entities, as already noted in Smith and Mark (2001a).

Overall, first, the results suggest that the word “geographic” denotes a coherent and familiar domain. Across cultures and contexts, participants frequently listed large natural features and objects associated with the Earth’s surface, such as *mountains, rivers, lakes, oceans, valleys, hills, plains, plateaus, deserts*, and *volcanoes* (Mark et al., 2010).

Second, they suggest that the geographic domain breaks down into different categories depending on the terms used in elicitation tasks, and this holds across contexts and cultures. While at first sight, results might indicate that just one folk ontology of the geospatial domain—modulated by task—exists, more subtle conceptual differences between US and Portuguese cultures might still have been hidden by methodological divergences across the studies. Moreover, assuming the existence of a universal ontology of the geo domain based on comparing two Western industrialized environments that are quite like each other would be premature (Majid, 2023). Such a conclusion appears even less straightforward if we frame this result considering the evidence on conceptual variability reviewed in the previous sections (see sections “6 Cross-linguistic and cross-cultural variability of geo concepts,” “6.1 The variability of geo concepts: evidence from free-listing tasks,” “6.2 The variability of geo concepts: evidence from ethnophysiographic methods,” and “6.3 Two paradigmatic cases of geographical variability: the concepts of *Landscape* and *Forest*”).

On a different note, some contributors tried to assess the effect of expertise on geo knowledge on its conceptualization. Giannakopoulou et al. (2013) performed a series of experiments similar to those in Smith and Mark’s (2001a,b) comparing Greek geography experts and non-experts. They found significant differences between the tasks but no substantial effect of expertise. A recent study by Purves et al. (2023) also failed to see such an effect. The authors compared the conceptualization of German and English-speaking experts and non-experts in geography on knowledge of 25 concepts related to water bodies (e.g., *sea, river, reef*) by collecting sensory, motor, and affective ratings. Their results showed that English and German experts’ and laypeople’s conceptualizations of water bodies broadly align—specifically in aspects concerning sensory and motor grounding—while expertise was less relevant for explaining the few differences found across samples. Interestingly, the results also suggested a considerable discrepancy between the meanings assigned to the terms “geography” and “geographic” by academics and laypeople. This also indirectly emerged from another study by Wartmann et al. (2014). Using typical ethnographic methods, the authors investigated the linguistic encoding of plant features of Madidi, an indigenous Spanish-speaking population of Bolivia. Specifically, the authors compared Madidi plant categories with the current standard scientific classification of plant terms. Their findings evidenced a gap between the Madidi native conceptualization and scientific taxonomies. Specifically, how the Madidi population classifies vegetation encompassed more numerous and complex terms and reflected more fine-grained differences than those included in the encyclopedia. For instance, when Madidi added the Spanish suffix “-al” to a plant name, it became a generic landscape term. This aspect was not at all considered in the traditional scientific classification (Wartmann et al., 2014).

Although we did not find substantial references on that, a variability in geo conceptualization might exist not only between laypeople and experts but also across scientific disciplines—that is to say, between experts from different fields—as hypothesized and partially documented in section “2 What do geographical and geopolitical concepts refer to?”

The studies reviewed indicate that the conceptualization of geographic entities varies due to factors beyond linguistic and cultural backgrounds. These include experimental setups and levels of expertise, which reflect differences in tasks and the individuals tested. While evidence on linguistic and cultural factors appears strong across languages and cultures (e.g., Mark et al., 1999, 2001; Smith and Mark, 2001a,b; Pires, 2005), findings regarding expertise are more controversial (e.g., Giannakopoulou et al., 2013; Purves et al., 2023; but also see Wartmann et al., 2014).

### 8.2 The influence of places and their familiarity on geo conceptualization

In contrast with the “geographical stability” highlighted by studies performed in Mark’s lab, other evidence shows that the geo conceptualization of laypeople is highly variable depending on context. Specifically, Wartmann et al. (2015) found that words listed for geographic categories varied depending on where the task was performed. In their study, participants were recruited directly from three Swiss study sites, two located in the mountains and one in a city park. Participants were asked to answer the question “What is there for you in a landscape?” and to produce all the words that came to their minds. Results revealed that the place where the task was performed influenced listed words and their order: participants produced similar terms when they were in similar landscapes and different terms when they were in different places. Notably, differences were most visible in ranking the most salient terms. For instance, the cognitive salience of “Berge” (*mountains*) considerably differed from the mountain study sites, where it was the most salient term, to the city park, where it ranked 6^th^. Nonetheless, the three study sites shared a consistent set of basic terms, and half of the 30 most salient terms were listed by all participants. Interestingly, the task location also affected the memory search strategies of participants when it came to listing words. Indeed, individuals often combine two methods, first producing visible elements of the surrounding landscape and then using the memory of a familiar place to name terms for that landscape.

On top of that, a study by Williams et al. (2012) demonstrated that familiarity with a place also plays a pivotal role in its conceptualization. Their study was designed to account for the effects of the context on categorization processes when disentangled from cultural and linguistic influences (see sections “6.2 The variability of geo concepts: evidence from ethnophysiographic methods” and “6.3 Two paradigmatic cases of geographical variability: the concepts of *Landscape* and *Forest*”). The authors recruited participants inhabiting two different areas of Portugal, i.e., the Lousã region in the north of Portugal, characterized by mountains covered in natural and plantation forests, and the Odemira region in the south of Portugal, an area mainly consisting of lowlands and small undulating hills. Participants from the Lousã and Odemira study sites were asked to watch videos displaying the two regions, name all the landforms they could identify, and provide place names of any locations they recognized. Results revealed differences in the landform vocabulary size and content between the two groups, with familiarity mostly accounting for these differences. Participants used more landform terms to describe the most familiar landscapes. In addition, the landform vocabulary content was more detailed when it concerned prominent landscape features in which participants lived. Finally, the number of scenes people recognized positively correlated with the number of landform terms they used to describe the videos in both groups. This suggests that place recognition—indicated by knowing a place name for the video scene location—promoted a more detailed landform categorization. In other words, places’ familiarity, along with the peculiarity of the context, seems to be a further driver for categorization. More specifically, the mental maps people form for familiar places might drive the identification of landform categories through associated knowledge of what happens at a given location.

## 9 Hierarchical levels of categorization of the geo domain

This final section deals with the information people store about the places they inhabit and how this content is organized in semantic memory, i.e., a kind of human long-term deposit that conserves information about the world (Tulving, 2011; Reilly et al., 2023). We will illustrate studies inspired by the classical theory of hierarchical levels (Rosch and Mervis, 1975; Rosch et al., 1976, 1978). According to this proposal, semantic information is organized in a three-level conceptual hierarchy. Superordinate concepts (e.g., *animal*) are maximally distinctive but not very informative; subordinate concepts (e.g., *bulldog*) are very informative but not very distinctive. Finally, intermediate, basic level concepts (e.g., *dog*) would be the most frequently used, as they maximize informativeness and distinctiveness, as well as represent those containing a greater amount of stored information.

Patton (1995) extended Rosh’s view to the geo domain by proposing a three-level hierarchy in which geo-information is stored at a subordinate, basic, or superordinate level. The nodes in the subordinate level correspond to actual places a person has experienced, and the basic-level nodes represent more abstract categories of places. For example, when we visit a location like *Rome*, we learn about that specific city (subordinate level) and the basic level category *city*, which is more abstract. At the highest hierarchical level (i.e., superordinate) is the most abstract spatial concept, i.e., the category of *place*.

Along these lines, Lloyd et al. (1996) were interested in assessing the *amount* of information deposited at the superordinate, basic, and subordinate levels for geo categories. They asked participants to list as many characteristics, activities, or parts they associated with a particular geo term as possible. Characteristics were defined as “attributes that could be used to describe something”; activities were defined as things that “reflect responses or behaviors”; parts were defined as “things you can see and interact with” (Lloyd et al., 1996, pp. 187–188). The sample was divided into 11 groups: 5 groups were asked to generate terms (characteristics, activities, or parts) for the subordinate level categories: “home county”, “home region”, “home state”, “home city”, and “home neighborhood”. Other 5 groups were asked to generate terms for the basic-level categories: “country”, “region”, “state”, “city”, and “neighborhood”. Finally, a third group was asked to generate terms for the superordinate category “place”. The sample consisted of 630 students from introductory geography classes at the University of South Carolina, Columbia, and the University of North Carolina, Greensboro. In line with Rosch’s view (Rosch and Mervis, 1975; Rosch et al., 1976, 1978), results showed a substantial increase of information between the superordinate and basic levels and relatively little difference between the basic and subordinate levels for all three types of information. Therefore, the main argument regarding the amount of information stored at each hierarchy level from Rosh’s theory seems to be strongly supported by geo categories.

Tversky and Hemenway (1983) also conducted several experiments on this topic. In such experiments, they used “indoors” and “outdoors” as superordinate categories. Basic level categories were “home”, “school”, “store”, and “restaurant” for the indoor scene and “park”, “city”, “beach”, and “mountains” for the outdoor scene. The subordinate categories were specific cases like “elementary school” for “school” or “Midwestern city” for “city”. The first set of experiments provided 210 participants with both photographs and verbal descriptions of environmental scenes at three levels of abstraction and had them give lists of attributes, activities, and parts appropriate for each scene. Results indicated that scenes representing superordinate categories (“indoor” and “outdoor”) shared very few attributes, activities, and parts, suggesting that the scenes were very distinctive. Basic-level scenes (e.g., “beach”, “mountains”, “homes”, “school”) were less distinctive than superordinate categories, i.e., they shared many attributes, activities, and parts, but also more informative, as participants could list significantly more features. Scenes for more specific categories at the subordinate level (“elementary school” and “lake beach”) did not share significantly more properties than scenes representing basic-level categories—suggesting they were only slightly less distinctive than basic-level scenes. Because the number of activities, attributes, and parts listed for subordinate scenes only slightly exceeded that for the basic-level scenes, they were also somewhat more informative than basic-level scenes. Among the various types of items listed by participants, Tversky and Hemenway (1984) argued that parts proliferated in participants’ listings for basic-level categories. Still, few parts were listed for superordinate categories. This aligns with previous literature on hierarchical levels of categorization (Rosch et al., 1976, 1978; Hemenway, 1981; Murphy and Smith, 1982). In this regard, Tversky and Hemenway suggested that members of basic-level categories can be distinguished by their parts. Still, members of subordinate categories share parts and differ in other attributes. In the second set of studies (Tversky and Hemenway, 1983, 1984), the authors asked participants to provide labels for photographs of scenes and to complete sentences such as “The Kingstons furnished their ______ with furniture they built themselves”. Results revealed that participants preferred basic-level terms. Indeed, they used more frequently basic level terms when labeling photographs of scenes and when completing sentences describing activities performed in scenes, even though more specific or more general terms would have been appropriate too. Thus, the conceptual hierarchical level preferred in communication corresponds to the level determined to be basic under perceived appearance and perceived behaviors. In conclusion, the authors showed that knowledge about scenes and objects has a hierarchical organization in which an intermediate level of abstraction is preferred in cognition, behavior, and communication measures.

Overall, findings confirm that, like other semantic domains, geo entities are stored according to the hierarchical levels of categorization proposed by Rosch and colleagues (Rosch and Mervis, 1975; Rosch et al., 1976, 1978). They, therefore, highlight the necessity to consider the difference between concepts’ hierarchical levels in efficient governmental communication on sustainability, referred to the geo domain.

## 10 Conclusion

For decades, the conceptualization of geo—i.e., geographical and geopolitical—entities has been at the center of debate among scientists—especially those in the geographical and psychological fields—which is still ongoing. At the core of the discussion, there is the question of whether the categorization can be considered universal or if linguistic and cultural differences influence it. While some approaches defended the idea of homogeneity of geo classification (e.g., Council of Europe, 2000), most of the positions illustrated in this review strongly support the thesis of relativity (non-universality) of these categories. Indeed, numerous pieces of evidence showed that geo categories consistently vary across contexts, cultures, and languages (e.g., Burenhult and Levinson, 2008; Turk and Stea, 2014) and are even more variable than other semantic domains (e.g., Battig and Montague, 1969; van Overschelde et al., 2004; van Putten et al., 2020). Importantly, geo-meaning is also influenced by many other potential factors, such as people’s level of expertise (e.g., Giannakopoulou et al., 2013; Purves et al., 2023; but see also Wartmann et al., 2014), the kind of implemented task (e.g., Mark et al., 1999, 2001; Smith and Mark, 2001a,b; Pires, 2005), living environments along with their perceived familiarity (e.g., Williams et al., 2012; Wartmann et al., 2015). So, our review indicates that geo concepts are a highly flexible class of words.

This evidence suggests that geo concepts might possess a *hybrid* character. Indeed, although they usually refer to concrete entities—i.e., referents that can experience through actions and senses (e.g., Brysbaert et al., 2014)—as typical concrete concepts, their high variability makes them similar also to more abstract words (for linguistic/cultural variability of abstract concepts, see e.g., Borghi et al., 2018a,b, 2019a,b; Borghi, 2019, 2022; Borghi and Mazzuca, 2023; for variability in expertise, see e.g., Croijmans et al., 2020; Villani et al., 2022).

The malleability of this special domain can be partially explained by the fact that geographical and geopolitical features are not pre-segmented by nature but depend on how we look at them to categorize their exemplars. This is a direct consequence of their intrinsic nature: their referents, like *valleys* and *coasts*, or human transformations of them, like *cities* or *ports*, are not merely located in space but are also typically parts of the Earth’s surface, which is a continuous surface.

Investigating geographical and geopolitical concepts might add further and essential evidence to laypeople’s overall perception of the natural environment. This might be helpful for the definition of a domain ontology and inform the improvement of geographical information systems, like *Google Earth*, that assist in everyday human activities, such as navigation. It might also contribute to raising consciousness about how people experience the environmental damages caused by recent political contrasts and the climate change emergency (Baquiano and Mendez, 2015, 2016; Malt and Majid, 2023) and, vice versa, how pre-existing representation of places they inhabit might affect the (lack of) ecological and political actions for contrasting them.

Crucially, assessing whether and to which extent geo meaning varies according to cultural and linguistic aspects can provide a conceptual basis for identifying and implementing more idiosyncratic governmental policies on these topics that can allow communicating more effectively and thus reach as many people as possible. For instance, promoting effective policies for sustainable development (as those, for instance, described in the United Nations 2030 Agenda - United Nations, 2015) requires effective communication, which, in turn, involves a deep comprehension of how people of different countries conceptualize their world and can be motivated to change it.

The knowledge of the various ways laypeople conceptualize the geographical and geopolitical domains and the recognition that languages, cultures, people’s expertise, and the environments where they live deeply influence their conceptualizing represents, in our view, a critical piece allowing the promotion of a better world.

## Author contributions

IF: Conceptualization, Resources, Writing – original draft, Writing – review and editing. CF: Writing – review and editing. CM: Writing – review and editing. AB: Conceptualization, Funding acquisition, Writing – review and editing.
